# Nutrition Management in Critically Ill Children: A Scoping Review of Current Practices and Outcome Measures in the Pediatric Intensive Care Unit

**DOI:** 10.3390/nu18081284

**Published:** 2026-04-18

**Authors:** Isabella R. Purosky, Terry Griggs, Chana Kraus-Friedberg, Mara L. Leimanis-Laurens

**Affiliations:** 1 College of Human Medicine, Michigan State University, 15 Michigan St NE, Grand Rapids, MI 49503, USA; puroskyi@msu.edu (I.R.P.); griggst2@msu.edu (T.G.); 2 MSU Libraries, 366 W. Circle Drive, East Lansing, MI 48824, USA; krausfri@msu.edu; 3 Pediatric Critical Care Unit, Helen DeVos Children’s Hospital, Corewell Health, 100 Michigan Street NE, Grand Rapids, MI 49503, USA; 4 Pediatrics & Human Development, College of Human Medicine, Michigan State University, East Lansing, MI 48824, USA

**Keywords:** pediatric intensive care, critically ill children, clinical nutrition, enteral nutrition, parenteral nutrition, feeding tolerance, nutritional adequacy, malnutrition, pediatric intensive care outcomes, scoping review

## Abstract

**Background/Objectives:** Nutrition is essential to outcomes in critically ill children; however, optimal timing, route, and composition of feeding remain uncertain. Prior studies demonstrate considerable variability in study design, patient populations, and outcome measures, limiting comparability. This review synthesizes international pediatric intensive care unit (PICU) nutrition studies evaluating timing, route, and content of nutritional interventions and summarizes associated clinical outcomes and nutritional adequacy. **Methods:** A comprehensive scoping review was conducted using the PICOS framework. PubMed and Embase databases were searched for studies published between 2015 and 2025 enrolling critically ill children ≤21 years old admitted to PICUs. Eligible studies assessed timing (early vs. late enteral nutrition), nutritional composition, or feeding route (enteral vs. parenteral). Screening and full-text review were performed independently by two reviewers using Covidence, with discrepancies resolved by a third reviewer. Quality assessment used STROBE. The protocol was registered with PROSPERO. **Results:** Of 652 identified records, 30 studies met inclusion criteria. Studies were conducted primarily in the United States (27%), with additional contributions from Spain and Brazil (10% each) and several other countries. Study designs included randomized controlled trials (27%) and observational studies (73%). Interventions examined feeding route (14%), nutritional content (38%), and timing (48%). Frequently reported outcomes included feeding intolerance or adverse events, duration of mechanical ventilation, time to nutrition goals, PICU length of stay, mortality, and nutritional adequacy. **Conclusions:** The contemporary PICU nutrition literature demonstrates persistent heterogeneity in practice and outcomes. This review identifies ongoing gaps in timing, delivery, and adequacy of nutritional support.

## 1. Introduction

Malnutrition remains a prevalent and pressing concern in the pediatric intensive care unit (PICU). A recent systematic review and meta-analysis encompassing 4331 participants reported that more than one in three critically ill children were malnourished during their PICU stay [1]. The etiology of malnutrition in this population is multifactorial, involving a complex interplay between increased metabolic demands, difficulty in accurately estimating energy expenditure, and inadequate substrate delivery at the bedside [1,2,3]. Together, these factors contribute to both protein and caloric deficits throughout the course of critical illness [3].

The early foundational literature, including the narrative review “Nutrition Management of the Critically Ill Pediatric Patient,” highlighted the clinical importance of nutrition support in critically ill children while also underscoring the substantial challenges inherent to studying it rigorously [4]. Pediatric critical care nutrition research is frequently limited by multifarious patient populations, rapidly changing clinical trajectories, ethical concerns regarding withholding or delaying nutrition support, and difficulty isolating the independent effects of nutrition within complex critical illness physiology. These realities restrict the feasibility of large randomized controlled trials and have resulted in a literature base that relies heavily on observational and retrospective data to inform practice [4].

Within this context, a pivotal randomized controlled trial published in *The New England Journal of Medicine* in 2016, commonly referred to as The Early versus Late Parenteral Nutrition in the Pediatric Intensive Care Unit (PEPaNIC) trial, provided rare high-level evidence by evaluating the timing of supplemental parenteral nutrition in critically ill children. The trial demonstrated that delaying parenteral nutrition during the first week of critical illness reduced infection rates, shortened duration of mechanical ventilation, decreased PICU length of stay, and lowered health care-associated complications without worsening nutritional status [5]. Although highly influential, the PEPaNIC trial was not included in this review because patient recruitment occurred largely before our predefined inclusion window. Subsequent follow-up analyses of the PEPaNIC cohort have examined longer-term outcomes, reporting no detrimental developmental effects up to four years after PICU admission among children in whom early parenteral nutrition was withheld, while also highlighting that developmental impairments remain common in PICU survivors overall [6,7]. The present review intentionally focuses on more recent studies to reflect contemporary practice patterns and evolving guideline recommendations. Nevertheless, its findings continue to frame discussion in pediatric critical care nutrition and remain foundational to current research directions [5].

Since PEPaNIC, increasing research attention has focused on refining nutrition strategies in the PICU, including timing, route, composition, and adequacy of delivery. Much of this work has been observational or retrospective, which has allowed for evaluation of real-world practice while also reflecting the ethical and practical challenges of conducting randomized controlled trials in critically ill children. Several important studies, including contributions from our group, have characterized metabolic perturbations in critically ill children with multi-organ dysfunction, demonstrated alterations in the gut microbiome among critically ill infants, and documented real-world gaps between prescribed and achieved energy and protein delivery in children supported with extracorporeal membrane oxygenation [8,9,10,11]. Collectively, these studies illustrate both the physiologic complexity and practical barriers that continue to shape pediatric nutrition care, while reinforcing the need for sustained systematic evaluation.

In 2025, the American Academy of Pediatrics (AAP) released updated recommendations emphasizing the importance of early identification and individualized nutritional management in critically ill children [2]. These recommendations include screening and assessing all children on admission to identify those at high risk of malnutrition, personalizing energy and protein delivery goals, and avoiding overzealous macronutrient administration during the acute phase of illness. When available, indirect calorimetry should be used to measure energy expenditure accurately [2]. Enteral nutrition (EN) remains the preferred route, ideally initiated within 24–48 h of admission and advanced using stepwise algorithms to achieve prudent goals within the first week. Parenteral nutrition (PN) should be used judiciously, serving as a supplement or alternative when EN is contraindicated or inadequate, and early PN initiation should be avoided. Continuous monitoring for intolerance, feeding complications, and refeeding syndrome in high-risk patients is also advised [2]. In select clinical situations, specialized nutritional therapies such as ketogenic diet therapy may also be administered parenterally in the intensive care setting, with emerging consensus-based guidance recommending stepwise initiation, careful monitoring, and transition back to enteral feeding as soon as clinically appropriate [12].

Recent reviews in pediatric critical care nutrition have predominantly focused on isolated components of nutritional management, including topics like early enteral nutrition, protein intake, or the impact of nutritional status on clinical outcomes [13,14,15,16,17,18,19,20]. Many of these studies are limited to single intervention types, specific patient populations, or narrowly defined clinical questions, and several are narrative in nature without systematic evaluation of the included studies [13,14,15,16,17,18,19,20,21,22]. As a result, there remains a lack of comprehensive synthesis across the full spectrum of nutritional strategies in the pediatric intensive care unit. This scoping review addresses this gap by integrating evidence across multiple domains, including timing, route, and nutritional content, to provide a broader and more cohesive understanding of current practices, variability in definitions and outcomes, and areas requiring further investigation.

## 2. Methods

### 2.1. Protocol Registration

**Figure 1:**
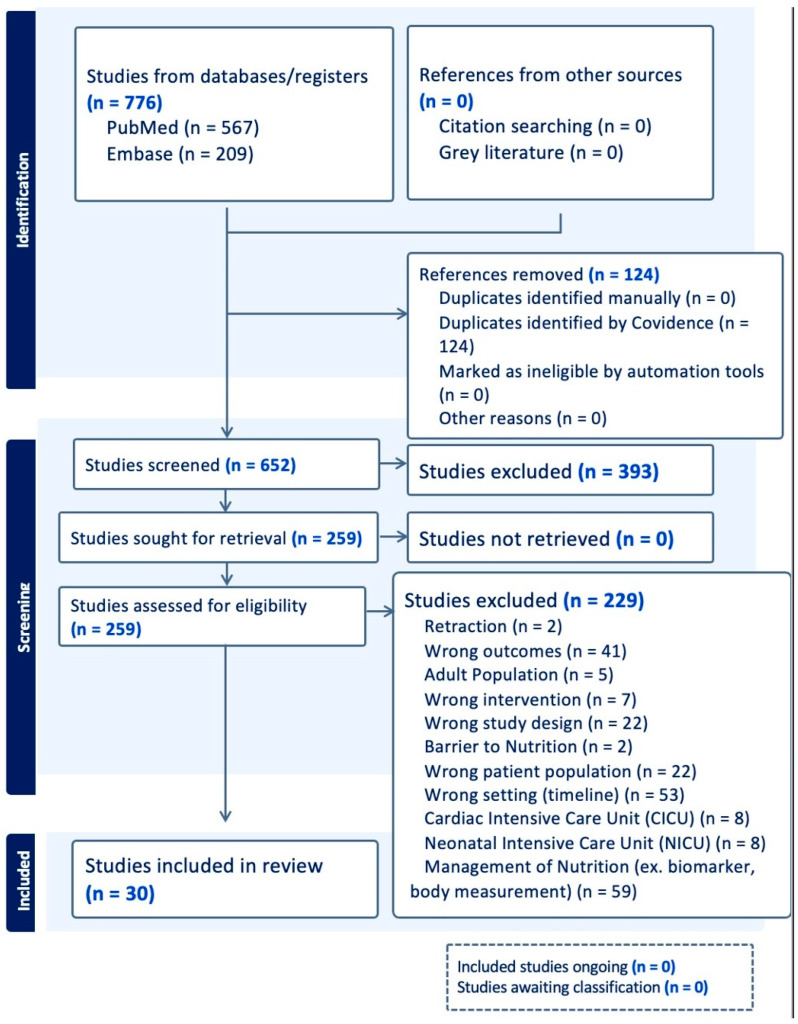
PICU nutrition PRISMA chart via Covidence.

This scoping review was registered in the International Prospective Register of Systematic Reviews (PROSPERO) (Registration No. CRD420251163395) and was conducted in accordance with the Preferred Reporting Items for Systematic Reviews and Meta-Analyses (PRISMA) guidelines (Figure 1). The review addressed the following research questions: “Do current studies provide evidence for an optimal nutritional strategy in critically ill pediatric patients?” according to PICOS (Patient/Population (P): Critically ill pediatric patients within a PICU; Intervention (I): Nutritional interventions evaluating timing of initiation (e.g., early vs. late enteral nutrition); route of feeding (e.g., enteral vs. parenteral, intermittent vs. continuous), or nutritional composition (e.g., protein content and formula type); Comparison (C): Not applicable; Outcome (O): Clinical and nutritional outcomes, including mortality, PICU length of stay, duration of mechanical ventilation, time to achieve nutrition goals, nutritional intake and adequacy, and feeding intolerance or adverse events; and Study Design (S): RCTs, cohort, observational, and ex vivo.

### 2.2. Search Strategy and Inclusion/Exclusion Criteria

Searches were performed in PubMed and Embase between 20 March and 16 April 2025. The search strategies for PubMed and Embase are detailed in (Supplemental Table S1). Studies were limited to articles published in English between 1 January 2015 and 16 April 2025. Studies were included if they reported results on pediatric, critically ill, human patients in a PICU who were assessed for nutritional status and/or received nutritional interventions.

Case reports, survey studies, quality improvement studies, practice guidelines, review articles, opinion papers, gray literature, animal studies, and letters were excluded. Studies of humans who were over the age of 18, not critically ill, not PICU patients, or were not directly assessed for nutritional status and/or were not receiving nutritional interventions were excluded as well. Studies of pre-term or low-birth-weight infants were also excluded. Further, as seen in Figure 1, ongoing studies and studies awaiting classification were also excluded.

### 2.3. Article Screening and Data Abstraction

The study selection process followed PRISMA guidelines (Figure 1). All records were screened independently by two reviewers using Covidence (Veritas Health Innovation, Melbourne, Australia). Titles and abstracts were initially screened for eligibility, followed by a full-text review of studies meeting inclusion criteria. When disagreements arose at either the title/abstract or full-text screening stage, they were resolved through discussion between the two reviewers (I.R.P., T.G.). If consensus could not be reached, a third reviewer (M.L.L.-L.) was adjudicated to achieve a final agreement.

The initial search yielded 652 results after duplicates were removed. During title and abstract screening, 393 records were excluded. A total of 252 articles underwent full-text review, of which 222 were excluded for predefined reasons, including irrelevance to the research question or classification into excluded subcategories such as nutritional assessment (*n* = 59), neonatal intensive care unit (NICU) populations (*n* = 8), and cardiac intensive care unit (CICU) populations (*n* = 8).

During full-text review, additional exclusions were made by reviewer consensus (I.R.P., M.L.L.-L., and T.G.) based on the study recruitment period. As a result, a total of 30 articles were analyzed fully [23,24,25,26,27,28,29,30,31,32,33,34,35,36,37,38,39,40,41,42,43,44,45,46,47,48,49,50,51,52]. Studies in which the majority of patient enrollment occurred prior to 2015 were excluded from data extraction to ensure relevance to contemporary pediatric critical care nutrition practices. I.R.P. and T.G. independently conducted data extraction, with each article extracted by a single reviewer (15 articles each). Extracted data were categorized based on intervention type, including timing, route, and nutritional content. To ensure consistency, both reviewers performed quality control (QC) on a subset of two random articles extracted by the other reviewer, with inter-rater reliability (IRR) calculated at 80%. No automation tools were used, and discrepancies identified during QC were resolved through discussion to achieve 100%.

A qualitative synthesis approach was selected due to differences in study design, interventions, and outcome reporting. No data conversions or imputations were performed, and all data were extracted as reported in the original studies. Effect measures were not predefined, as results were not quantitatively synthesized and were instead reported descriptively across studies. Formal subgroup or meta-regression analyses to explore sources of variability were not performed. Sensitivity analyses were not conducted due to the absence of quantitative synthesis.

The studies by Solana et al. (2021) [43] and Solana et al. (2023) [44] represented secondary analyses derived from the same patient population. These studies evaluated different cohorts based on distinct inclusion and exclusion criteria. Two studies by Winderlich et al. (2024) [50] were conducted using the same patient population, with one of the studies being a secondary analysis of the other. The secondary analysis by Winderlich et al. (2024) [49] represented a subset of the initial study and had distinct inclusion and exclusion criteria. The two studies by Martinez et al. (2022) [35] and Martinez et al. (2023) [36] were conducted in distinct analytical cohorts despite sharing the same primary author. Martinez et al. (2022) [35] represents a secondary analysis of the Pediatric International Nutrition Study (PINS) cohort previously described by Bechard et al. (2021) [24] and applied different eligibility criteria, resulting in a separate analytic sample. Martinez et al. (2023) [36] evaluated a different intervention focus and did not represent overlap in patient populations.

### 2.4. Quality of Reporting Assessment

Strengthening the Reporting of Observational Studies in Epidemiology (STROBE) is designed for observational studies, which 22 (73%) out of 30 articles met. The articles that were randomized control trials (RCTs) were reviewed regardless with the understanding that the portions of the checklist regarding study design were omitted. Two reviewers (I.R.P., T.G.) scored each manuscript following the STROBE 22-item checklist electronically on Covidence to assess the quality of the reporting of the studies we included. The following ratings were utilized per checklist item: high risk, low risk, or unclear bias. Any discrepancies were analyzed by a third reviewer (M.L.L.-L.) and if a consensus still could not be made, it was made by a discussion between all three reviewers (I.R.P., T.G., and M.L.L.-L.).

### 2.5. Bibliometric Analysis

Vosviewer provides network visualization and density visualization, which form clusters and links between items (i.e., author or keyword associations). The color of an item is determined by clusters, and the lines represent the links (https://www.vosviewer.com/). Maps were created using bibliographic data (database files, CSV. or nbib. exported from PubMed). Co-author and co-occurrence (using Medical Subject Headings MeSH keywords) analysis was done using Vosviewer 1.6.20 for visualization and map bibliometric indicators which were developed in the Java programming language [53]. Additional visualizations were performed including both the income of the country classification and primary author.

## 3. Results

### 3.1. Overview

**Figure 2:**
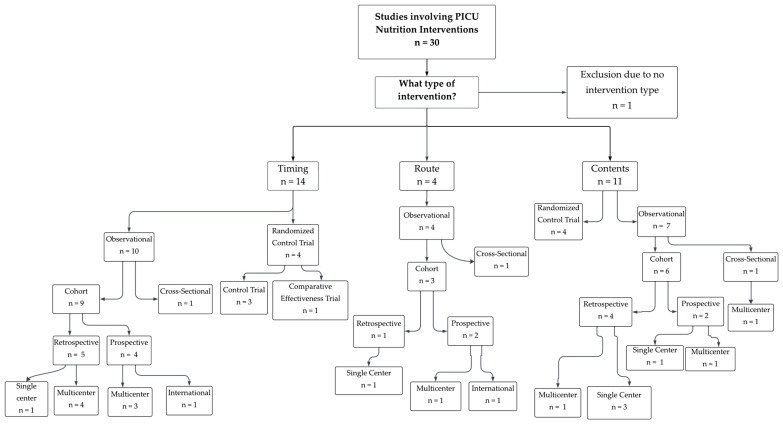
Flowchart of 30 publications isolating study design.

A total of 30 studies were included (Figure 2). The median publication year was 2022–2023. The median age of participants across studies was 17.5 months, with reported patient ages ranging from 2 days to 18 years. The median sample size was 99.5 participants (range: 18–1844). Two studies (7%) were conducted in the Middle East/North Africa and two (7%) in Oceania (Australia and New Zealand), while four studies (13%) were conducted in Asia, five (17%) in Latin America, and seven (23%) in Europe. Eight studies (27%) were conducted in the United States. Based on World Bank income classifications, fifteen studies (50%) were conducted in high-income countries, eleven (37%) in upper-middle-income countries, three (10%) in lower-middle-income countries, and one study (3%) included a mixed population from high- and upper-middle-income countries (Supplemental Figure S1). Twelve studies reported receiving funding (40%), twelve studies reported no funding (40%), and the remaining studies did not specify funding status, but were likely unfunded. Abstracted data was summarized in Table 1.

**Figure 3:**
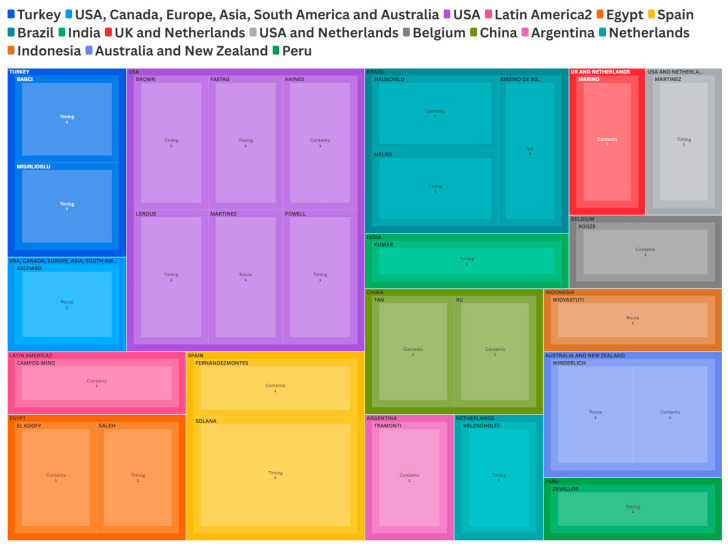
Visual stratification of intervention type by countries and authors.

Study designs included eight RCTs (27%) and 22 observational studies (73%). The included observational studies consisted of two cross-sectional studies, ten retrospective cohort studies, and eight prospective cohort studies. Of the 30 included studies, one was excluded from intervention-specific analyses due to the article being broad with no primary focus on a specific intervention despite meeting our inclusion criteria. Among the remaining studies, four (13%) examined routes of feeding interventions, 11 (37%) examined nutrition content interventions, and 14 (47%) examined timing-related interventions. A visual configuration of intervention type, primary author and country is summarized in Figure 3. Across all studies, reported outcomes included time to achieve goals in 12 studies (40%), nutritional intake in 13 studies (43%), and nutrition adequacy, mechanical ventilation duration, and feeding intolerance or adverse events in 14 studies each (47% each). PICU mortality and PICU length of stay were even more frequently reported, appearing in 19 (63%) and 23 (77%) studies, respectively. Individual study characteristics and outcomes are presented descriptively in Table 1, Table 2, Table 3 and Table 4. Quantitative synthesis, subgroup analyses, and sensitivity analyses were not performed due to differences in study design, interventions, and outcome reporting. The potential for reporting bias was considered, particularly given the likelihood of preferential publication of positive findings. Certainty of evidence was not formally evaluated.

### 3.2. Bibliometric Analysis Using Vosviewer

**Figure 4:**
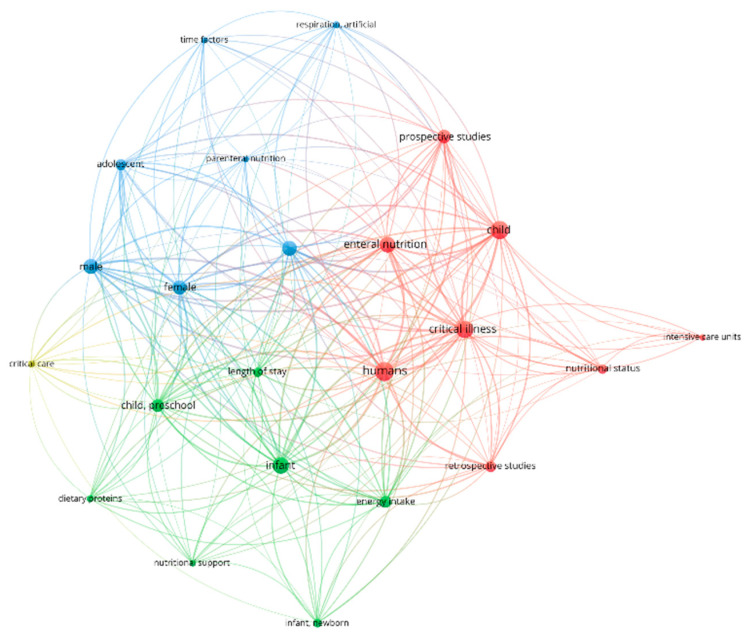
Minimum occurrence of three MeSH keywords (23 keywords met these criteria) using Vosviewer.

A minimum number of occurrences was set at three MeSH keywords; 23 met these criteria (Figure 4). If the minimum occurrence was set at only two MeSH keywords to determine the links, 28 keywords met these criteria (Supplemental Figure S2). Top MeSH keywords included “infant”, “humans”, “critical illness”, and “child”. Looking at keywords allows for a high-level summary of the main topics from the last decade.

**Figure 5:**
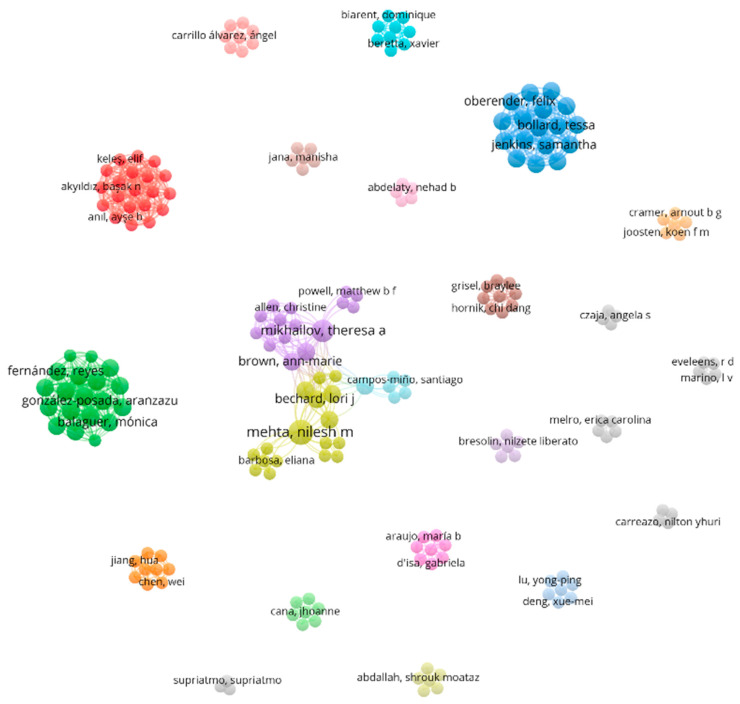
Network analysis where authors had a minimum of one co-citation, consisting of 42 authors (Vosviewer).

From a total of 202 authors, we included authors that had a minimum of one co-authoring citation (42 met this criteria) (Figure 5). Network analysis where authors had a minimum of two documents consisted of 25 authors (Supplemental Figure S3). Co-authorship analysis refers to an evaluation and analysis of co-authored articles and is considered a substantial indicator to determine leading countries or institutions to look at collaborative trends and main authors [54].

### 3.3. STROBE

Articles were screened by two independent reviewers using the STROBE guidelines (22-point checklist; IRR 88.6%) to assess methodological and reporting quality. The mean overall quality score of the included studies was 91.7%, with all manuscripts meeting at least 16 of the 22 STROBE criteria. All initial discrepancies were resolved through consensus. While STROBE is not a formal risk of bias assessment tool, it was used to evaluate study quality across key reporting domains. A formal risk of bias tool was not applied given the range of study designs included.

### 3.4. Timing Intervention

Among the 14 included studies, designs consisted of one (7%) cross-sectional study, four each of both randomized controlled trials and prospective cohort studies (28.5%), and five (36%) retrospective cohort studies. Outcomes reported across studies included achievement of nutrition goals and nutritional intake in five studies (36%), feeding intolerance or adverse events (AEs) as well as nutrition adequacy noted in six studies each (43%), time to achievement of nutrition goals in eight studies (57%), mortality and duration of mechanical ventilation (MV) in ten studies (71%), and PICU length of stay in all studies (100%) (Table 2).

Several studies reported measures of illness severity or mortality risk. Four studies reported Pediatric Logistic Organ Dysfunction (PELOD), seven studies reported Pediatric Risk of Mortality (PRISM-III), and five studies reported VIS scores. Eight studies (57%) incorporated more than one type of severity or mortality risk scoring, and eight studies (57%) utilized the Pediatric Index of Mortality (PIM) specifically (Table 2).

**Table 2:** Timing intervention type (*n* = 14).

First Author Last Name and Year	Intervention Specifics	Outcomes Investigated	Additional Measurements
Baǧci 2018 [23]	Early initiated feeding vs. early reached target enteral nutrition	PICU mortality PICU LOS Reached nutrition goal Nutrition adequacy Time to reach goal for nutrition Feeding intolerance	PIM2 Minimum arterial pH and base excess Maximum arterial lactate Maximum blood glucose Estimated energy requirements
Brown 2022 [25] ^1^	Bolus vs. continuous feeds	PICU LOS PICU mortality Nutrition adequacy Time to achieve goal feeds Feeding intolerance Mechanical ventilation duration	PIM2 OSI
Fastag 2025 [28]	EEN vs. LEN	PICU LOS PICU mortality Time to goal feeds Mechanical ventilation duration	ISS PIM2 PRISM-III VIS Associated barriers to initiation of EEN Opioid total daily dose
Kumar 2024 [32]	Continuous vs. intermittent tube feeding	PICU LOS PICU mortality Time to reach targets Feeding intolerance Mechanical ventilation duration	Glucose variability (hypoglycemia < 60 mg/dL, hyperglycemia > 180 mg/dL) PELOD and pSOFA Serum potassium Maximum and minimal inotropic score in 24 h
Leroue 2017 [33] ^2^	EEN vs. LEN	PICU LOS Nutrition adequacy Time to goal EN rate Nutrition goal achieved (within 72 h) Adverse events Frequency of EN interruptions greater than 6 h	Mode/length of NIPPV PRISM III
Martinez 2022 [35]	Intermittent vs. continuous enteral nutrition	PICU LOS PICU mortality Nutrition adequacy Time to achieve 60% adequacy Achieved nutrition goal?	PIM 1 and 2 PRISM 2 and 3
Melro 2020 [37] ^3^	EEN vs. LEN	PICU LOS PICU mortality Nutrition adequacy Nutrition intake Mechanical ventilation duration	PIM2 VIS
Misirlioglu 2025 [38]	Intermittent vs. continuous enteral feeding	PICU LOS Nutrition intake Reaching target calories Mechanical ventilation duration Feeding intolerance Adverse events	VIS PIM2 PRISM-III PELOD Metabolic/electrolyte abnormalities Blood gas and blood sugar levels
Powell 2022 [39] ^4^	EEN vs. non-EEN	PICU LOS PICU mortality Mechanical ventilation duration Probability of PICU discharge Percent enteral and parenteral energy	OI OSI PELOD-2 Days from PARDS diagnosis
Saleh 2023 [42]	Early PN vs. late PN	PICU LOS Nutritional intake Mechanical ventilation days Feeding intolerance Adverse events	PRISM pSOFA Vasoactive infusion (day) Liver dysfunction in ICU Cholestasis in ICU Hypoglycemia
Solana 2021 [43]	EEN vs. LEN	PICU LOS Nutrition intake Mechanical ventilation duration Adverse events	PRISM-III Complications in patients on EN vs. PN Indirect calorimetry (IC)
Solana 2023 [44]	24-EEN vs. 24-LEN and 48-EEN vs. 48-LEN ^1^	PICU LOS Nutrition intake Nutrition adequacy Time to reach goal Mechanical ventilation duration Adverse events	PRISM-III VIS Need for CRRT NIV
Veldscholte 2023 [47]	Intermittent vs. continuous feeds	PICU LOS PICU mortality Nutrition intake Adverse events Feeding intolerance Feasibility	Daily 3-b-hydroxybutyrate, BHB Hyperglycemic events Ketone levels PIM3 PELOD
Zevallos 2024 [52]	EEN vs. LEN	PICU LOS PICU mortality Mechanical ventilation duration Time to achieve goal Frequency of enteral interruptions Adverse events	Vasoactive drug usage PIM2 Lactate level PaO2/FiO2

LOS = Length of stay; ECI = enteral caloric intake; EER = estimated energy requirements; OI = oxygenation index; OSI = oxygen saturation index; ERTEN = early reached target EN; VIS = vasoactive inotrope score; pSOFA = Pediatric Sequential Organ Failure Assessment scoring; pREE = predicted resting energy expenditure; PARDS = Pediatric Acute Respiratory Distress Syndrome. Special patient groups: ^1^ mechanically ventilated patients only; ^2^ children requiring Non-invasive Positive Pressure Ventilation (NIPPV); ^3^ PICU patients with respiratory insufficiency; ^4^ PARDS3.5. route intervention.

Timing-related interventions included early initiation of enteral feeding versus early achievement of target enteral nutrition in one study (7%), early PN versus late PN in another study (7%), intermittent (bolus) feeding versus continuous feeding in five studies (36%), and early enteral nutrition (EEN) versus late enteral nutrition (LEN) in seven studies (50%). Definitions of EEN and LEN varied across the seven studies evaluating this comparison: one study (14%) examined a 72 h threshold, another study (14%) examined both 24 h and 48 h thresholds, two studies (29%) used a 48 h cutoff, and three studies (43%) used a 24 h cutoff (Supplemental Table S3). Feeding intolerance was explicitly defined in six studies. Of these, five studies defined feeding intolerance, as seen in Supplemental Table S3, based solely on patient signs and symptoms, while one study incorporated both clinical signs/symptoms and predefined protocol-specific criteria. Gastric residual volume (GRV) measurement was included as a criterion in five of the six studies that defined feeding intolerance (Supplemental Table S3).

Nutrition guidelines referenced in Supplemental Table S3 included ASPEN/SCCM 2009 in two studies, The European Society for Paediatric and Neonatal Intensive Care (ESPNIC) 2020 in five studies, and The American Society for Parenteral and Enteral Nutrition/Society of Critical Care Medicine (ASPEN/SCCM) 2017 in nine studies. Several studies additionally reported using country-specific or institutional nutrition guidelines rather than international consensus guidelines, or a combination of multiple guideline sources. Energy expenditure or energy requirement estimation was reported using varying terminology across studies, including energy expenditure, basal metabolic rate, and energy requirements. Estimation methods included one study (7%) utilizing both equations, three studies (21%) utilizing neither, with the World Health Organization (WHO) equation being utilized in four studies (29%), and the Schofield equation being used in six studies (43%).

Designs included in this intervention category were single-center retrospective cohort and single-center cross-sectional studies in one study each (25% each), and two multicenter prospective observational cohort studies (50%).

The routes compared included EN versus EN + PN, EN versus PN versus EN + PN, postpyloric EN versus gastric EN, total parenteral nutrition (TPN) versus partial parenteral nutrition (PPN) with EN, and EN adequacy without a comparison route in one study each (25% each).

### 3.5. Route Intervention

Reported outcomes across these studies included mechanical ventilation duration, percentage of caloric objective achieved, and protein intake in one study each (25% each) and PICU length of stay and time to achieve nutrition goals in two studies each (50% each). PICU mortality and nutrition adequacy were the most frequently reported outcomes, appearing in three studies each (75% each) (Table 3).

Of the additional measurements reported, mortality risk or severity of illness was assessed using validated scoring systems in all four studies (100%). Specifically, one study (25%) utilized the PELOD-2 and two studies (50%) utilized the PIM2 (Table 3).

**Table 3:** Route intervention type (*n* = 4).

First Author Last Name and Year	Intervention Specifics	Outcomes Investigated	Additional Measurements
Bechard 2021 [24]	Enteral nutrition vs. enteral + parenteral nutrition	PICU mortality Nutrition adequacy Nutrition intake Time to reach goal	PIM2
Martinez 2023 [36]	Postpyloric vs. gastric enteral nutrition	PICU LOS PICU mortality Nutrition adequacy Mechanical ventilation days Time to reach goal	PIM2
Widyastuti 2024 [48]	TPN vs. PPN (PN + EN)	PICU LOS Nutrition adequacy Nutrition intake Time to reach goal Achieved goal?	PELOD-2
Winderlich 2024 [50]	Enteral nutrition adequacy	PICU LOS PICU mortality Nutrition adequacy	Inotrope usage Principal admission diagnostic category ECMO usage

Route intervention definitions reported in Supplemental Table S4 varied across studies and were guided by both international and institutional standards. ASPEN and/or Society of Critical Care Medicine (SCCM) guidelines were utilized in two studies (50%), while the European Society for Paediatric Gastroenterology, Hepatology and Nutrition (ESPGHAN), National Health and Medical Research Council (NHMRC), Institute of Medicine (IOM), and World Health Organization (WHO) guidelines were each referenced in one study (25% each). Local or institutional protocols were reported in two studies (50%).

Early enteral nutrition was defined in one study (25%) as achieving 25% of prescribed energy targets within the first 48 h of PICU admission. Nutrition goals were defined as achieving ≥60% of prescribed energy and protein targets in two studies (50%). Reasons for enteral nutrition interruption were described in two studies (50%) and included fasting for procedures, diarrhea, chylothorax, worsening clinical status, lack of enteral access, or formula unavailability. Feeding or enteral nutrition intolerance was defined in three studies (75%) and was primarily characterized by clinical signs and symptoms including abdominal distension, abdominal discomfort, emesis, and diarrhea (Supplemental Table S4). 

### 3.6. Content Intervention

Studies in the content intervention type addressed a broad range of nutrition composition questions across distinct PICU populations, including enterally fed patients, parenterally supported patients, and disease-specific cohorts (Table 4). As presented, these studies would not be eligible for further sub-analysis. Designs within this category included one multicenter point prevalence study, multicenter prospective observational cohort study, multicenter retrospective cohort study, single-center prospective cohort study, and parallel randomized controlled trial (9% each), and three RCTs and single-center retrospective cohort studies (27% each). Interventions included comparisons of high fat with low carbohydrates; low- vs. high-dose MTEI-I; short peptides; high-protein-enriched, protein-enriched, and standard diets; peptide-based nutrient-dense enteral feeds; standardized nutritional support protocols; and descriptive evaluations of characteristics of enteral nutrition prescribed in one study each (9% each). Mixed-lipid versus pure soybean oil-based lipid emulsions and polymeric versus oligomeric or semi-elemental enteral formulas were represented in two studies (18% each) (Table 4).There was one study (9%) focused on pediatric patients with major burns, and two studies (18%) focused on mechanically ventilated children.

**Table 4:** Content-intervention-type specific nutrition contents (*n* = 11).

First Author Last Name and Year	Nutrition Contents
Campos-Miño 2023 [26]	Standard enteral feed
El Koofy 2019 [27]	Infants younger than 4 months of age were fed a ready-made formula with fat representing 50% of nonprotein calories (Infatrini, Nutricia) For those older than 4 months of age, high-fat enteral feeds were formulated by adding olive oil to blended food Isocaloric high-fat, low-carbohydrate enteral diet = 50% fat, 30% carbohydrates Standard isocaloric diet = 25% fat, 55% carbohydrates Reasons for feeding interruption = severe respiratory distress, gastrointestinal bleeding, paralytic ileus, poor adherence of PICU staff to feeding regimens were recorded, gastrointestinal intolerance
Fernández Montes 2023 [29]	Standard diet = 1.7 g/dL protein, 7.4 g/dL carbohydrate, 3.4 g/dL lipids, 67 energy (kcal) Protein-enriched diet = 2.7 g/dL protein, 10.3 g/dL carbohydrate, 5.4 g/dL lipids, 100 energy (kcal) High-protein-enriched diet = 5.1 g/dL protein, 10.5 g/dL carbohydrate, 5.5 g/dL lipids, 110 energy (kcal)
Haines 2023 [30]	4-OLE = soybean oil/MCT/olive/fish oil lipid
Hauschild 2019 [31]	1 g of polymeric whey protein = 3.6 kcal, 97%—0.88 (100% whey protein), 0 carbs (g), 0 lipids (g), 5.5 mg sodium, 12 mg potassium, 2.4 mg phosphorus 1 g oligomeric whey protein = 3.2 kcal, 82%—0.80 (100% whey protein), 0.08 carbs (g), 0.05 lipids (g), 6.5 mg sodium, 0 mg potassium, 0 mg phosphorus
Marino 2019 [34]	Peptide nutrient energy-dense enteral feed = 100 kcal and 2.6 g protein per 100 mL
Rooze 2020 [41]	100 mL classic semi-elemental diet = 65.9 kcal, 1.8 g proteins, 3.5 g lipids, 6.8 g carbohydrates, 18.3 mg sodium, 190 mOsm/L 100 mL classic polymeric diet = 65.9 kcal, 1.3 g proteins, 3.5 g lipids, 7.3 g carbohydrates, 17.3 mg sodium, 150 mOsm/L 100 mL hypercaloric semi-elemental diet = 100.4 kcal, 1.8 g proteins, 4.8 g lipids, 12.5 g carbohydrates, 18.3 mg sodium 100 mL hypercaloric polymeric diet = 100.4 kcal, 1.3 g proteins, 4.8 g lipids, 13 g carbohydrates, 17.3 mg sodium
Tan 2021 [45]	MTEI-(I) = zinc (249 μg/mL), copper (20.1 μg/mL), manganese (1 μg/mL), selenium (2 μg/mL), fluorine (F, 57 μg/mL) and iodine (I, 1 μg/mL) Low-dose MTEI-(I) = 1 mL/kg/d, and Group B Low-dose MTEI-(I) = 2 mL/kg/d, up to a maximum dose of 15 mL/d
Tramonti 2018 [46]	Lactose-free enteral formula as primary nutrition with adjustment to hydrolyzed formula if intolerance occurs Nonprotein calorie:nitrogen ratio of 100:1 Supplemental PN with glucose and amino acids when EN is insufficient Lipid-free PN initially with a maximum glucose flow of 5–7 mg/kg/min and amino acids at a rate of 30 g/L. In the case of exclusive PN, by day 7, lipids should be added at a rate of 0.5 g/kg/day and increased to 1 g/kg/day after 24 h if triglycerides are <250 mg/dL
Winderlich 2024 [49]	Formula = any commercial nutrition product provided to children via oral or enteral route Oral nutrition support = any prescribed oral intake with the intention of providing increased energy and/or protein Increased energy and protein density through enteral and oral nutrition support = any enterally administered or orally consumed formula or feed prescribed for the provision of additional energy and protein Types of enteral nutrition prescribed = breast milk, formula, donor milk Specifics of feed types: Standard = expressed breast milk or infant formula Increased density = expressed breast milk fortified with infant formula powder or macronutrient modules Specialized formulas = condition-specific, including peptide-based, renal-specific, and modified-fat formulas
Xu 2025 [51]	Short-peptide enteral nutrition formulations = Peptamen, Nestle Whole-protein enteral nutrition formulations = Nutrison, Nestle

Reported outcomes included time to reach energy and protein targets, discharge rate, and extubation rate in one study (9% each); duration of mechanical ventilation and protein intake in three studies (27%), feeding tolerance or intolerance in four studies each (36% each); and energy/calorie intake, nutrition adequacy (protein and/or calories) in six studies (55% each). Both PICU length of stay and PICU mortality were reported in eight studies each (73% each) (Table 5).

Additional metabolic, biochemical, and anthropometric measurements were frequently reported. Arterial blood gases, assisted minute ventilation, bilirubin, metabolomics, trace elements, phosphorous, and vitals were reported in one study (9% each); complete blood count, creatinine, liver function tests, inflammatory/metabolic markers, oxygenation index, and triglycerides were all assessed in two studies (18% each); energy expenditure and nitrogen balance in three studies (27%); serum protein or serum protein changes in five studies (45%); and anthropometrics were assessed in eight studies (73%). Severity of illness or mortality risk was evaluated using PELOD or PIM3 in one study (9% each); PCIS, PRISM, and RACHS-1 in two studies (18% each); and PIM2 in five studies (45%) (Table 5).

Reported guidelines and definitions in Supplemental Table S5 highlight that Chinese nutrition society guidelines were used in one study individually (9%). SCCM guidelines and guidelines from a collective of European and Chinese nutrition societies including ESPGHAN, ESPEN, ESPR, and CSPEN were applied in two studies (18% each); while WHO and local or hospital-based clinical practice guidelines were reported in three studies (27% each). ASPEN guidelines were utilized most frequently with representation in six studies (54%) (Supplemental Table S5).

Operational definitions related to nutritional targets, tolerance, and malnutrition were described in ten studies (91%), while one study (9%) did not include these definitions. Protocol-specific definitions including feasibility and feed advancement rates were defined in one study and two studies, respectively (9% and 18%). Malnutrition definitions were provided in four studies (36%), while energy or nutrient goals and feeding intolerance were defined in five studies (45% each) (Supplemental Table S5).

## 4. Discussion

Across the included studies, three major categories of nutritional interventions emerged: timing, route, and content of feeds. Although intervention strategies varied, some definitional consistency was observed (Supplemental Tables S3–S5). The Schofield and WHO (FAO/WHO/UNU) equations remain the most commonly applied tools within our review for estimating resting energy expenditure in critically ill pediatric populations; however, both were originally developed from healthy cohorts and rely primarily on demographic and anthropometric variables such as age, sex, weight, and height [55,56,57]. Although their widespread use reflects practicality and historical familiarity, their accuracy in the PICU setting is limited. Existing evidence suggests that these equations predict measured REE within ±15% in only about half of critically ill children and rarely achieve the clinically desirable ±10% precision threshold [56,57]. Consistent with this uncertainty, current ASPEN/SCCM guidelines permit the use of Schofield or WHO equations without added stress factors when indirect calorimetry is unavailable, acknowledging both their accessibility and inherent limitations [57]. More recent data indicate that alternative models may outperform traditional formulas in select populations; for example, a systematic review identified the Meyer equation as demonstrating favorable predictive accuracy among mechanically ventilated, critically ill children, underscoring the need for continued development and validation of population-specific REE prediction tools [58].

This scoping review highlights the substantial heterogeneity within the current literature on pediatric critical care nutrition. Variation was noted across study design, guideline utilization, and geographical distribution. Despite this variability, there was relative homogeneity in economic context, with 90% of the included studies being conducted in high-income or upper-middle-income countries. Only three studies were conducted in lower-middle-income countries, and no studies originated from low-income countries. Additionally, only approximately one-third of the studies reported receiving dedicated funding. This lack of representation from lower-resource settings has important implications. Health system capacity, resource availability, and access to PICUs differ substantially across income settings, and these factors likely influence both feasibility and effectiveness of nutrition interventions [59]. Limited PICU availability in low-income countries or settings has been associated with disproportionately high childhood mortality, with nearly 90% of deaths in children under five occurring in low- and middle-income countries [59]. Therefore, the predominance of high-resource settings in the available literature introduces an inherent bias and limits the global generalizability of current evidence. Ultimately, as emphasized by a systemic review in 2024 by Kortz et al., more resources are necessary to promote child health research and high-quality data collection in low-income and middle-income countries to further determine priority setting and resource allocation [59,60]. Preliminary bibliometric analysis revealed that co-authorship, which looks at the relationship between authors, was limited to a few authors, spanning the summarized articles included. This type of analysis can provide insight into collaborations and predominant authors and their subsequent organizations.

Within timing-related interventions, EEN consistently demonstrated clinical benefit within the subsequent conclusions of these studies; however, there was substantial variability in how “early” and “late” feedings were defined, with most studies using either a 24 h or 48 h threshold [23,28,33,37,43], one study evaluating both timeframes [44], and another study evaluating a 72 h threshold [52]. Although the overall evidence favors earlier initiation regardless of cutoff, this variability limits comparability and underscores the need to determine whether a specific threshold confers greater benefit or whether a more individualized, physiology-guided timeline is warranted [23,28,33,37,43,44,52]. Diversity was also evident in the assessment of feeding intolerance. Despite limited supporting evidence, GRV monitoring remains widely used in pediatric intensive care units as seen in our review, with most definitions incorporating GRV alongside gastrointestinal symptoms such as distention, vomiting, and diarrhea [23,25,38,42,47]. However, GRV has demonstrated poor correlation with clinically meaningful outcomes in critically ill children and has not been reliably associated with aspiration risk, delayed gastric emptying, or the ability to advance enteral nutrition as emphasized by ASPEN 2017 guidance [57]. Contemporary guidelines therefore caution against routine reliance on GRV and acknowledge its uncertain role, recommending more symptom-based, protocolized approaches to intolerance assessment in the PICU [57,61,62].

Across route-based nutritional intervention studies, results were varied in intervention comparisons, outcome selection, and definitional frameworks. Studies compared EN alone, PN alone, combined EN + PN strategies, postpyloric versus gastric feeding, or adequacy of enteral nutrition without a comparator. Outcomes ranged from nutrition adequacy and time to nutrition goals to PICU mortality and length of stay. Differences in guideline utilization resulted in inconsistent definitions of nutrition targets, feeding intolerance, early nutrition, and reasons for feed interruptions, limiting cross-study comparability. Measurement approaches included estimated energy requirements and severity of illness scores; however, the differences between scoring models and energy requirement calculations likely contributed to inconsistent associations between route of nutrition and clinical outcomes. Notably, no randomized controlled trials examined the route of nutrition within the PICU, reflecting the ethical and practical challenges of conducting RCTs in critically ill children. The lack of controlled trials in pediatric populations suggests that observational designs will continue to dominate the literature unless ethical and safe trial designs can be established.

Studies evaluating nutritional content demonstrated variability in feed composition, with studies consisting of high-fat, low-carbohydrate formulas; peptide-based formulas; macronutrient and micronutrient enrichment; lipid emulsions, and nutrition protocol strategies. Outcomes consisted of nutritional intake and adequacy, feeding tolerance, biochemical markers, PICU length of stay and mortality. Guideline adherence differed markedly across studies, with use of multiple versions of ASPEN/SCCM; WHO, European and Chinese society recommendations; and institution-specific protocols. Guideline diversity led to nonuniform definitions of nutrition goals, feeding intolerance, malnutrition, and feasibility. Although these nutrition societies often draw inspiration from the same collection of evidence, the resulting guidelines differ. For instance, the 2016 *New England Journal of Medicine* trial [5] on delayed parenteral nutrition informed both ASPEN and ESPEN guidelines; however, ASPEN recommends earlier feeding for patients who are malnourished at admission, whereas ESPEN does not include this exception, contributing to potential differences in international practice. Furthermore, several studies examined highly specialized populations (e.g., burn, neonatal, mechanically ventilated or TPN-dependent patients), in which nutritional interventions and formula composition are expected to differ, contributing to methodological differences and limiting direct comparison across studies. Additional measurements were highly varied, incorporating energy expenditure, nitrogen balance, anthropometrics, laboratory biomarkers, vitals, respiratory status, and various illness severity scores, which were used inconsistently across studies. As a result of the variability across the nutritional content domain, interpretation of the effectiveness of specific nutritional compositions is restricted, and comparison across studies remains challenging. However, the nutritional content domain shows a greater willingness to employ RCTs in pediatric research compared to route-based designs, which may facilitate the standardization of nutritional recommendations. Future studies that examine comparable patient populations and standardized nutritional endpoints will be essential for determining the clinical impact of specific nutrient compositions in critically ill children.

### Limitations

This review has several limitations. Only English-language publications were included, introducing potential language bias, and publication bias is possible given the tendency for positive findings to be published. Only PubMed and Embase were used for the literature search. Findings cannot be used to recommend policy/practice. Studies from low-income countries were underrepresented, likely reflecting differences in research infrastructure, access to pediatric intensive care, and financial barriers to publication, which may limit generalizability. The predefined publication window excluded studies published prior to 1 January 2015 or after 16 April 2025, and therefore may not have captured earlier foundational work or the most recent evidence. Heterogeneity in study design, intervention definitions, and outcome measures limited direct comparability across studies, and many included studies may have been underpowered, requiring findings to be interpreted descriptively rather than as guidance for clinical practice or policy.

An additional limitation of this review is the limited consideration of micronutrient composition, particularly in the context of parenteral nutrition. While this review focused on macronutrient delivery, timing, and route of administration, emerging evidence suggests that essential micronutrients, including trace elements and vitamins, play a critical role in metabolic function and may influence the utilization and efficacy of macronutrients in critically ill pediatric populations [63,64]. Variability in micronutrient supplementation across studies was not systematically evaluated and may have contributed to differences in reported outcomes. Future studies should incorporate standardized assessment and reporting of micronutrient provision to better elucidate their role in pediatric critical care nutrition.

## 5. Conclusions

Collectively, these findings reinforce the clinical importance of early feeding while highlighting the need for harmonized timing definitions and modernization of intolerance assessment practices to improve comparability and optimize care. This disparity restricts the ability to draw definitive conclusions regarding optimal nutrition routes in the PICU and complicates translation into standardized clinical practice, underscoring the need for harmonized definitions, guidelines, and outcome measures in future route-focused research. This highlights the need for standardized guideline adoption and uniform definitions and outcomes to improve clinical applicability. To further this narrative, our group is in the process of doing a meta-analysis with articles that have similar outcome measurement choices to further expand upon this review topic.

## Figures and Tables

**Table 1:** Overall demographics of 30 publications involving nutritional interventions within PICUs.

PMID	First Author Last Name	Study Design	Intervention Type	Year	Country	Income Class	Age Ranges	Median Age (Months)	Sample Size	Funding (Y/N)
29278447	Baǧci [23]	Multicenter retrospective cohort study	Timing	2018	Turkey	Upper Middle	1 month to 16 years	4 ± 4.7 *	95	Y
34320161	Bechard [24]	Multicenter prospective observational cohort study	Route	2021	USA, Canada, Europe, Asia, South America and Australia	High/Upper Middle	1 month to 18 years	19.7	1844	Y
34881440	Brown [25]	Multicenter prospective randomized comparative effectiveness trial	Timing	2022	USA	High	1 month to 12 years	7.7	147	Y
37539965	Campos-Miño [26]	Multicenter point prevalence study ^1^	Contents	2023	Latin America ^2^	Upper Middle	1 month to 18 years	36.5	311	-
30999727	El Koofy [27]	Randomized control trial	Contents	2019	Egypt	Lower Middle	1 month to 12 years	12	51	-
39893842	Fastag [28]	Retrospective cohort study	Timing	2025	USA	High	1 month to 18 years	97	238	N
37344303	Fernández Montes [29]	Multicenter prospective randomized control trial	Contents	2023	Spain	High	1 month to 24 months	4	99	Y
37669048	Haines [30]	Single-center retrospective cohort study	Contents	2023	USA	High	1 month to 17 years	36	684	Y
29959852	Hauschild [31]	Randomized control trial	Contents	2019	Brazil	Upper Middle	1 month to 14 years 11 months and 29 days	3.7	25	N
38064124	Kumar [32]	Randomized control trial	Timing	2024	India	Lower Middle	1 month to 18 years	54	58	N
28816919	Leroue [33]	Single-center retrospective cohort study	Timing	2017	USA	High	>30 days old	24	562	-
30848864	Marino [34]	Multicenter retrospective cohort study	Contents	2019	UK and The Netherlands	High	<12 months old	2.6	53	Y
36306567	Martinez [35]	International prospective observational cohort study	Timing	2022	USA and The Netherlands	High	1 month to 18 years	15.6/16.9	1375	Y
36722708	Martinez [36]	Single-center retrospective cohort study	Route	2023	USA	High	<21 years	-	92	Y
32359758	Melro [37]	Prospective observational analytical cohort study	Timing	2020	Brazil	Upper Middle	1 month to 14 years	6	71	Y
39861429	Misirlioglu [38]	Prospective observational multicenter study	Timing	2025	Turkey	Upper Middle	1 month to 18 years	55/34	510	N
34961948	Powell [39]	Retrospective cohort study	Timing	2022	USA	High	2 weeks to 18 years	49.2	151	N
37921951	Ribeiro de Souza [40]	Single-center prospective cohort study	N/A	2023	Brazil	Upper Middle	One month of corrected age ^3^ to less than 15 years	9	108	N
31781932	Rooze [41]	Single-center retrospective cohort study	Contents	2019	Belgium	High	2 days to 6 years	0.82	100	N
34961948	Saleh [42]	Randomized control trial	Timing	2023	Egypt	Lower Middle	1 month to 16 years	18/18.5	140	N
33109454	Solana [43]	Multicenter prospective cross-sectional study	Timing	2021	Spain	High	1 month to 16 years	9.5	86	-
36268895	Solana [44]	Multicenter observational prospective study	Timing	2022	Spain	High	1 month to 16 years	8	68	-
34765482	Tan [45]	Parallel randomized control study	Contents	2021	China	Upper Middle	<18 years	**	40	Y
30016025	Tramonti [46]	Single-center prospective cohort study	Contents	2018	Argentina	Upper Middle	<16 years old	46.8	18	N
37478810	Veldscholte [47]	Randomized control trial	Timing	2023	The Netherlands	High	Term newborn to 18 years	4.8	140	Y
39816116	Widyastuti [48]	Single-center cross-sectional study	Route	2024	Indonesia	Upper Middle	28 days to 18 years	120	60	N
37984244	Winderlich [50]	Multicenter prospective observational cohort study	Route	2024	Australia and New Zealand	High	Less than or equal to 18 years	6	141	-
39037417	Winderlich [49]	Multicenter prospective cohort study	Contents	2024	Australia and New Zealand	High	Less than or equal to 2 years	***	84	N
39867286	Xu [51]	Single-center retrospective cohort study	Contents	2025	China	Upper Middle	Neonate ^4^	****	90	Y
32359758	Zevallos [52]	Retrospective cohort observational single center study	Timing	2024	Peru	Upper Middle	1 month to 17 years	19	370	N

* Mean age with standard deviation; ** range of included patients was between 29 days and 10 years old; *** 94% of patient population was ≤1 year of age. **** Mean age with standard deviation ^1.^ Single day within another retrospective cohort study (NutriPIC) (multicenter); ^2.^ Argentina, Brazil, Bolivia, Chile, Colombia, Cuba, Honduras, Guatemala, Mexico, Panama, Peru, Uruguay; ^3.^ 44 weeks of gestational age; ^4.^ mean age in months for control group: 3.6 ± 2.4 and for experiment group: 3.5 ± 2.5.

**Table 5:** Content intervention Type (*n* = 11).

First Author Last Name and Year	Intervention Specifics	Outcomes Investigated	Additional Measurements
Campos-Miño 2023 [26]	Evaluation of enteral nutrition delivery relative to guideline-based energy (Schofield equation) and protein (ASPEN ≥ 1.5 g/kg/day) target	PICU LOS Nutrition intake Nutrition adequacy	PIM3 Nutrition status (z-BMI and MUAC)
El Koofy 2019 [27]	Isocaloric high-fat, low-carbohydrate enteral diet vs. standard isocaloric diet	PICU mortality Nutrition adequacy Nutrition intake Discharge rate Duration of mechanical ventilation Feeding intolerance	Arterial blood gases Assisted minute ventilation Anthropometrics Prealbumin Triglycerides Albumin CRP CBC LFTs PIM-2 Predicted energy expenditure (WHO)
Fernández Montes 2023 [29]	High-protein-enriched diet vs. protein-enriched diet vs. standard diet	PICU LOS PICU mortality Nutritional intake Serum protein changes	Serum proteins Nitrogen balance Indirect calorimetry PRISM PELOD PIM2
Haines 2023 [30]	Switch from pure soybean oil-based LE (Intralipid, “IL”) to a mixed-lipid emulsion (4-OLE/ SMOFLipid)	PICU LOS PICU mortality Nutritional intake	Total time receiving lipids Change in LFTs
Hauschild 2019 [31]	Polymeric vs. oligomeric vs. control	Nutrition intake Nutrition adequacy	PIM2 Nutrition status (z-BMI and MUAC) Nitrogen balance Creatinine Phosphorus
Marino 2019 [34]	Peptide nutrient energy-dense enteral feed	PICU LOS PICU mortality Achievement of nutritional target Duration of mechanical ventilation Feasibility/tolerance	Gastric residual volume Anthropometric measurements Energy requirements PIM2 RACHS-1
Rooze 2020 [41]	Semi-elemental diet vs. polymeric diet (EN)	Nutrition intake Feeding intolerance	-
Tan 2021 [45]	Low-dose multi-trace element injection (MTEI-I) vs. high-dose MTEI-I administered with parenteral nutrition	PICU LOS Pediatric critical illness score (PCIS)	Metabolomic measurements Trace elements Anthropometrics Blood labs: CBC, LFTs, creatinine, bilirubin, albumin Vital sign changes
Tramonti 2018 [46] *	Nutritional support protocol	PICU LOS PICU mortality Nutrition adequacy Nutrition intake Time to reach goals Time to initiation of enteral feeding	Zinc and copper levels Vitamins A, E, D levels Prealbumin CRP levels Urine urea nitrogen Nitrogen balance
Winderlich 2024 [49]	Types of enteral nutrition prescribed	PICU LOS PICU mortality Nutrition adequacy **	Proportion of children ≤2 y receiving EN or PN
Xu 2025 [51]	Short-peptide enteral nutrition formulation vs. whole-protein enteral nutrition	Length of hospital stay PICU mortality Duration of mechanical ventilation Gastrointestinal intolerance	Resting energy expenditure Oxygenation index Ventilator-associated pneumonia incidence Nutritional risk screening (STRONGkids) Pediatric critical illness score (PCIS) Biochemical nutritional markers Anthropometrics Oxygenation index

Special patient groups: * Major burn patient. ** Among children offered oral intake: the proportion that received oral nutrition support and the extent of oral intake relative to estimated requirements.

## Data Availability

No new data were created; however, any additional information is available upon request.

## Supplementary Materials

The following supporting information can be downloaded at https://www.mdpi.com/article/10.3390/nu18081284/s1: Table S1: Search Criteria; Table S2: PICOS framework for included studies; Figure S1: Visual stratification of income of countries and authors; Figure S2: Minimum occurrence at two MeSH keywords (28 keywords met these criteria) using Vosviewer; Figure S3: Network analysis where authors had a minimum of two documents consisted of 25 authors (Vosviewer); Table S3: Timing Intervention Definitions (*n* = 14); Table S4: Route Intervention Definitions (*n* = 4); Table S5: Contents Intervention Type Definitions and Guidelines (*n* = 11).

## Abbreviations

AE	Adverse Event
AAP	American Academy of Pediatrics
ASPEN	American Society of Parenteral and Enteral Nutrition
CICU	Cardiac Intensive Care Unit
ECMO	Extracorporeal Mechanical Oxygenation
EEN	Early Enteral Nutrition
EN	Enteral Nutrition
GRV	Gastric Residual Volume
IOM	Institute of Medicine
IRR	Inter-Rater Reliability
LEN	Late Enteral Nutrition
MeSH	Medical Subject Headings
MV	Mechanical Ventilation
NHMRC	National Health and Medical Research Council
NICU	Neonatal Intensive Care Unit
PELOD	Pediatric Logistic Organ Dysfunction
PEPaNIC	The Early versus Late Parenteral Nutrition in the Pediatric Intensive Care Unit Trial
PICU	Pediatric Intensive Care Unit
PIM	Pediatric Index of Mortality
PN	Parenteral Nutrition
PPN	Partial Parenteral Nutrition
PRISMA	Preferred Reporting Items for Systematic Reviews and Meta Analyses
PRISM-III	Pediatric Risk of Mortality
RCT	Randomized Control Trial
REE	Resting Energy Expenditure
SCCM	Society of Critical Care Medicine
TPN	Total Parenteral Nutrition
VIS	Vasoactive–Inotropic Score
