# Supplementary material for: Nutrition Management in Critically Ill Children: A Scoping Review of Current Practices and Outcome Measures in the Pediatric Intensive Care Unit
Source: Nutrients. 2026 Apr 18;18(8):1284. doi: 10.3390/nu18081284 (PMC13118292; doi:10.3390/nu18081284)
Supplement: Supplementary file 1 [file nutrients-18-01284-s001.zip › nutrients-4237162-supplementary.pdf]

Supplemental:

Supplemental Table S1. Search Criteria

PubMed:

1	pediatric[tiab] OR child[tiab] OR pediatrics[tiab] OR children[tiab] OR "Pediatrics"[Mesh]
2	nutrition[tiab] OR nutritional[tiab] OR nutrients[tiab] OR "Nutritional Status"[Mesh]
3	intensive care[tiab] OR "critical care"[tiab] OR "critically ill"[tiab] OR ICU[tiab] OR "Critical Care"[Mesh] OR "Intensive Care, Neonatal"[Mesh] OR "Intensive Care Units, Pediatric"[Mesh]
4	1 AND 2 AND 3
5	4 NOT Meta-Analysis[pt] NOT Case Reports[pt] NOT Systematic Review[pt] NOT Review[pt] NOT Letter[pt] NOT Guideline[pt] NOT Practice Guideline[pt] NOT Editorial[pt]
6	Filters: (2015/01/01/2015: 2025/04/16[pdat]) AND (English[Filter])

Embase:

1	pediatric:ti,ab OR child:ti,ab OR pediatrics:ti,ab OR children:ti,ab OR pediatrics:de
2	nutrition:ti OR nutritional:ti OR nutrients:ti
3	'intensive care':ti,ab OR 'critical care':ti,ab OR 'critically ill patient' OR 'pediatric intensive care units':de
4	1 AND 2 AND 3
5	'article'/it
6	[humans]/lim
7	4 AND 5 AND 6
8	7 AND NOT 'review'/de NOT ('practice guideline'/exp OR 'practice guideline') NOT 'meta analysis'/de NOT 'case report'/de NOT 'systematic review'/de NOT 'nonhuman'/de
9	'adolescent':ag OR 'child':ag OR 'infant':ag OR 'newborn':ag OR 'school':ag OR 'young adult':ag
10	8 AND 9
11	10 AND [01-01-2015]/sd NOT [17-04-2025]/sd

Supplemental Table S2. PICOS framework for included studies

PICOS concepts	Criteria for scoping review
Participants	Critically ill children aged 0–21 years hospitalized in a pediatric intensive care unit (PICU)
Interventions	Nutritional interventions, including timing (e.g., early vs. late), route (enteral vs. parenteral), and nutritional content (e.g., macronutrient composition, formula type)
Comparisons	Not applicable
Outcomes	Clinical and nutritional outcomes, including mortality, PICU length of stay, duration of mechanical ventilation, time to achieve nutrition goals, nutritional intake and adequacy, and feeding intolerance or adverse events
Study Design	RCT's, cohort, observational, ex vivo

Supplemental Figure S1. Visual stratification of income of countries and authors

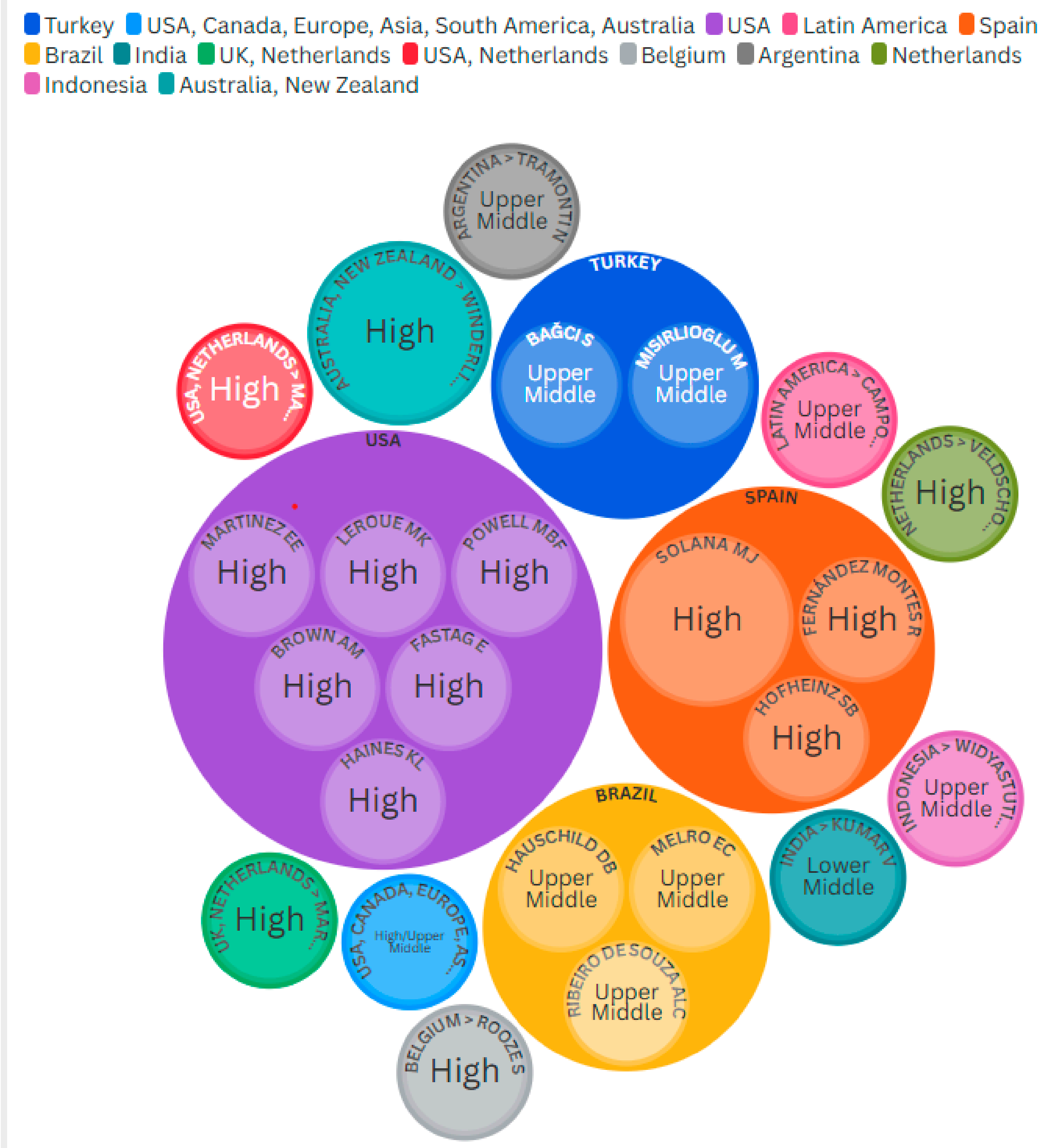

Supplemental Figure S2. Minimum occurrence at two MeSH keywords (28 keywords met these criteria) using Vosviewer

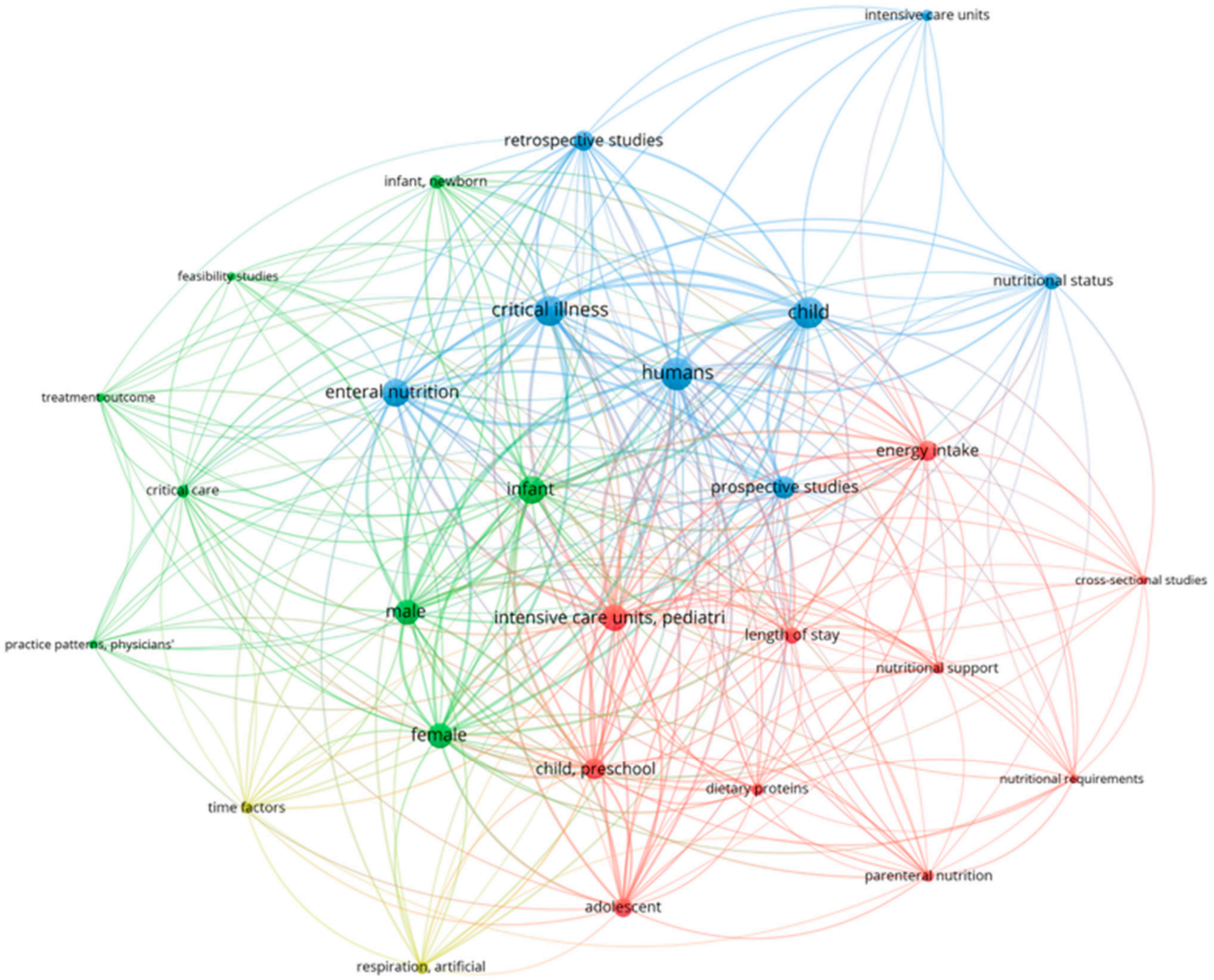

Supplemental Figure S3. Network analysis where authors had a minimum of two documents consisted of 25 authors (Vosviewer)

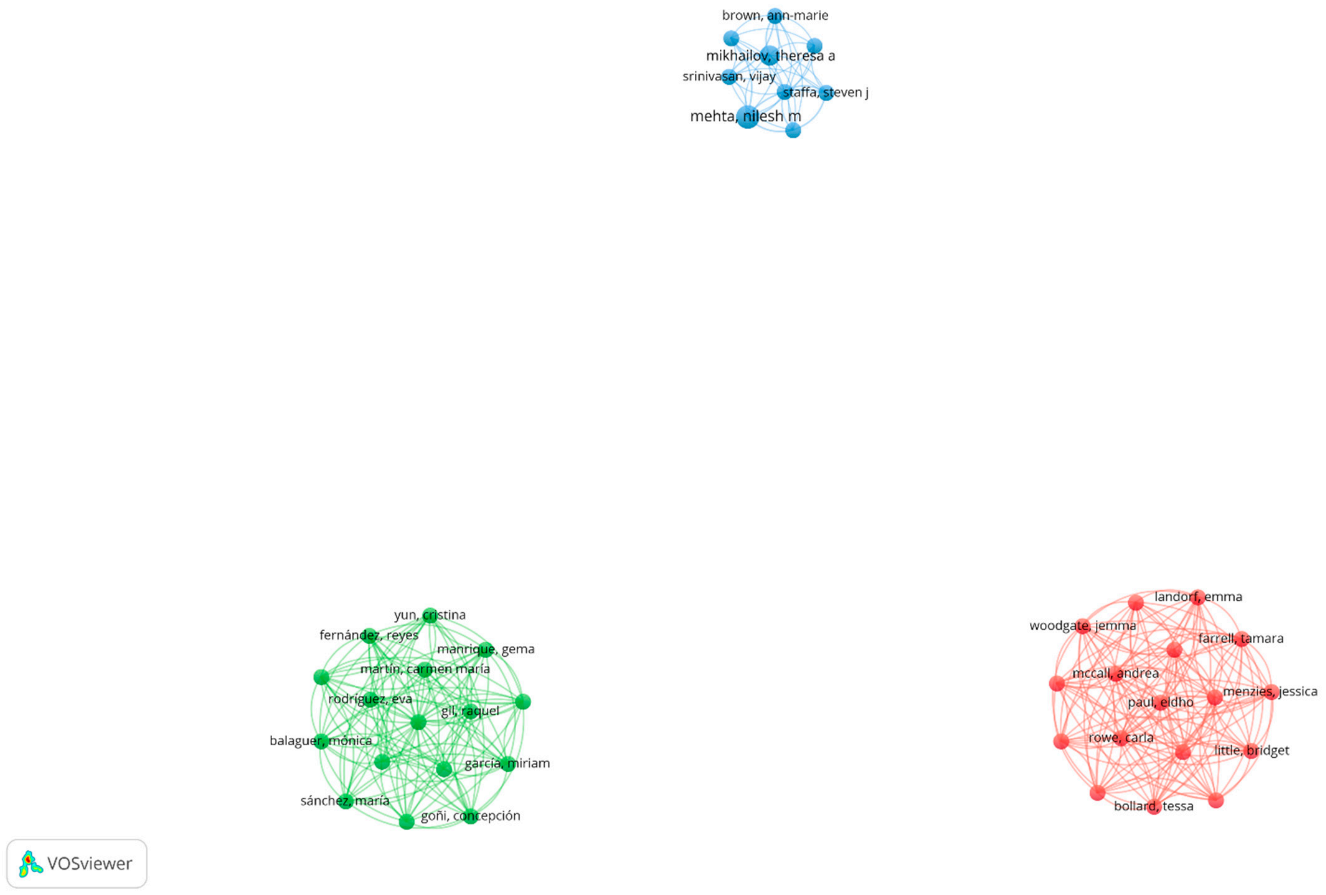

Supplemental Table S3. Timing Intervention Definitions (n = 14)

First Author Last Name and Year	Guidelines Utilized	Definitions
Bagci 2018	ASPEN 2017<br>ESPEN 2006<br>WHO	• **EIF** = any kind of tube feeding (gastric, duodenal, or jejunal) commenced within 24 hours of PICU admission<br>• **LIF** = any kind of tube feeding commenced 25-96 h following PICU admission<br>• **Feeding Intolerance Signs** = Gastric residual volume (GRV), bowel sounds, defecation, daily caloric intake, and 28-day mortality<br>• **Feeding Intolerance Biomarker** = Intestinal Fatty Acid Binding Protein (IFABP)<br>• **Estimated Energy Requirements Calculation** = World Health Organization (WHO) equation for calculating resting energy expenditure<br>• **Daily ECI Calculation** = Based on a study by Mikhailov et al.
Brown 2022	ASPEN 2017<br>USDA<br>WHO	• **Bolus Feeds** = 1 ml/kg as a 1-hour bolus<br>• **Continuous Feeds** = 1 ml/kg over 3 hours<br>• **Achieved Goal** = Achieving prescribed feed volume and energy density<br>• **Feeding Intolerance Signs** = emesis vs some combination of emesis, elevated GRV, or abdominal girth, based on site intolerance criteria<br>• **Energy needs in patients less than or equal to 24 months** = United States Department of Agriculture (USDA)<br>• **Energy needs in patients more than or equal to 25 months** = WHO equation
Fastag 2025	ASPEN 2017<br>WHO<br>CDC	• **EEN** = initiation of oral regular, soft, full-liquid diet or enteral tube feedings that provided at least 25% of daily caloric goals per ASPEN guidelines, as determined by the Schofield equation without adjusting for energy expenditures within 48 h of admission to the PICU<br>• **LEN** = any enteral feeding started >48 h after admission to the PICU or when less than 25% of caloric goal rate was achieved at 48 h.<br>• **Daily Caloric Goals** = calculated via Schofield Equation
Kumar 2024	ASPEN 2017<br>WHO	• **Intermittent Feeds**: received feeds at 2 ml/kg every 2 h (max. 50 ml/feed), and the rate was increased at 2 ml/kg every 4 h (max 50 ml) till the maximum fluid volume planned for the respective day was reached<br>• **Continuous Feeds**: received feeds at 1 ml/kg/h (maximum of 25 ml/h) and the rate was increased at 1 ml/kg/h (max. 25 ml/h) every 4 h till the maximum fluid volume planned for the respective day was reached<br>• **Target calories** = ≥79% of calculated calories as per WHO age specific equation after initiation of feeds)<br>• **Target protein** = 1.5g/kg as per ASPEN criteria<br>• **Feed Intolerance** = presence of any one of the features: vomiting, abdominal distension >10% increase from baseline girth, or diarrhea (≥3 episodes of loose stools)<br>• **Caloric Requirement** = calculated via WHO calculation
Leroue 2017	ASPEN 2009<br>Institutional NPO guidelines	• **EEN** = Enteral nutrition initiated within 24 hours of admission<br>• **Caloric Energy Goal** = ASPEN guidelines recommendation<br>• **Adequate EN** was defined as achieving greater than 67% of kcal and protein goals as an averaged, cumulative intake over the course of the PICU stay<br>• **Goal EN rate** = 100% of estimated kcal and protein requirements met via the hourly rate or bolus volume of tube feedings or through oral intake at a meal<br>• **Adverse events** = pneumonia not present at admission, intubation after enteral nutrition initiation, feeding tube misplacement<br>• **NIPPV** = heated high-flow nasal cannula (HHFNC), continuous positive airway pressure (CPAP), bi-level positive airway pressure (BiPAP), or average volume assured pressure support (AVAPS)<br>• **Malnutrition** = body mass index (BMI), z score of less than -1 or greater than 1. For children less than 2 years old, weight-for length z score was used instead of BMI
Martinez 2022	ASPEN 2017<br>WHO	• **Nutrition goal** = Achieve delivery of 60% energy and protein targets<br>• **Intermittent Feeding** = Any patients with interruptions in their feeds

First Author Last Name and Year	Guidelines Utilized	Definitions
		• **Reasons for EN interruptions** = Fasting for medical intervention or procedure, EN intolerance, a worsening clinical status, a lack of enteral access or nonavailability of an appropriate formula, or other reasons<br>• **Energy Target** = variety of methods including: the Schofield equation, WHO equation, local dietary reference intakes/RDAs, the patient's diet history, or other equations or guidelines
Melro 2020	ASPEN 2017<br>WHO	• **EEN group** = EN delivery (infant formula or human milk), via nasoenteric tube, or enterostomies tube, within the first 48 h of PICU admission<br>• **LEN** = initiation of EN 48 h after PICU admission<br>• **Protein requirement** = 1.5 g/kg/day based on ASPEN recommendations<br>• **Energy adequacy** = energy was determined adequate between 66 and 100%; protein was determined adequate ≥100%<br>• **Energy Requirement/basal metabolic rate** = WHO equations with no correction for stress factor
Misirlioglu 2025	Nutrition Working Group within the Turkish Society of Pediatric Emergency and Intensive Care<br>ESPNIC 2020	• **Continuous Feeds** = Initiated at a rate of 1 mL/kg/hour (up to a maximum of 25 mL/hour) with a feeding rate was increased by 1 mL/kg/h (max 25 mL/h) every 4 hours until target feeding was reached<br>• **Intermittent Feeds** = Patients under 6 months of age be fed every 3 hours, while patients 6 months of age should be fed every 4 hours. Feeding was initiated at 2 mL/kg/meal (max 50 mL/meal), and the volume was increased by 2 mL/kg/meal (max 50 mL/meal) after every three feedings, as tolerated, until the target feeding was reached<br>• **Feeding Intolerance Signs** = GI symptoms of feeding intolerance include vomiting/nausea, diarrhea (greater than 2mL/kg), abdominal distension, abdominal discomfort, constipation, aspiration, GI bleeding, and gastric residual volume of ≥ 150 mL or >3-5 mL/kg<br>• **Caloric Requirement Target** = Schofield equation
Powell 2022	ESICM 2017	• **ETP** = receiving 1.5 g/kg/day in the first 48 h from PARDS diagnosis in either enteral or parenteral forms<br>• **Basal Metabolic rate** = calculated utilizing Schofield Equation that was modified based on the stress of the current illness and interventions to resting energy expenditure
Saleh 2023	ASPEN 2009<br>ESPGHAN/ESPEN/ ESPR/CSPEN 2018<br>WHO	• **Early PN** = Both malnourished and well-nourished patients in this group started PN on the first day of admission<br>• **Late PN** = Malnourished patients started on the fourth day of PICU admission and Well-nourished were initiated on the seventh day<br>• **Feeding Intolerance =** The inability to digest enteral feedings associated to increased gastric residuals, abdominal distension, and/or emesis.
Solana 2021	Spanish Society of Pediatric Gastroenterology and Nutrition (SEGHNP); ASPEN 2017<br>ESPNIC 2020	• **EEN** = EN started within the first 48 h of admission<br>• **LEN** = EN started after 48 h of admission<br>• **Resting Energy Expenditure =** Determined by Schofield Equation with the exclusion of stress factors
Solana 2022	Spanish Society of Pediatric Gastroenterology and Nutrition (SEGHNP)<br>ASPEN 2017; ESPNIC 2020	• **24-EEN** = Early Enteral Nutrition that was administered within 24 hours;<br>• **24-LEN** = Late Enteral Nutrition that was administered after 24 hours;<br>• **48-EEN** = Early Enteral Nutrition that was administered within 48 hours;<br>• **48-LEN** = Late Enteral Nutrition that was administered after 48 hours<br>• **Resting Energy Expenditure** = Determined by Schofield Equation without the addition of stress factors
Veldscholte 2023	ESPGHAN/ESPEN/ESPR/CSPEN 2018<br>ASPEN 2017<br>ESPNIC 2020<br>Local nutritional protocol	• **Intermittent Feeding Group** = Nutrition was interrupted during an age-dependent overnight fasting period (neonates: 2am-10am; infants: 0am-10am; children: 10pm-10am)<br>• **Continuous Feeding Group =** Nutrition was administered day and night, with a maximum interruption period of 2 h excluding feeding interruptions due to clinical PICU care (e.g. interventions such as intubation and surgical procedures)<br>• **Feasibility** = defined as two conditions (1): a significant difference in the patients' highest daily ketone (BHB) levels during each overnight period, and (2): non-inferiority regarding daily caloric intake, examined using a two-part mixed-effects model with a predefined non-inferiority margin of 33% in an intention to treat analysis<br>• **Target Intake** = 100% pREE at the end of the first week and 130-200% pREE, depending on the child's weight<br>• **Insufficient intake** = <80% target intake; EN < two-third of prescribed daily target or EN withheld for≥ 48h or EN is not increased for ≥ 48h

First Author Last Name and Year	Guidelines Utilized	Definitions
		• **Criteria for Feeding Intolerance** = (1) Insufficient enteral intake. Excluding interruptions due to procedures or other medical reasons for not providing nutrition at target (e.g. fluid restriction, hemodynamic instability) (2a) GI symptoms such as large GRV, presence of vomiting ≥ 2 times in 24h period, or presence of diarrhea ≥ 4 times loose stool. (2b) Severe GI symptoms with concern for intestinal ischemia via melena or hematochezia<br>• **Resting Energy Expenditure** = predicted using the body weight-based or body weight and length-based Schofield Equation
Zevallos 2024	ESPNIC 2020	• **Full EN =** Administration of 80% of prescribed calories<br>• **Early EN** = Beginning of enteral feeding within 72 hours of admission to the PICU<br>• **Late EN** = Beginning enteral feeding after 72 hours following admission to the PICU<br>• **Adequate Protein Intake** = a minimum of 1.5 g/kg/d within the first three days

Supplemental Table S4. Route Intervention Definitions (n = 4)

First Author Last Name and Year	Guidelines Utilized	EN Definitions
Bechard 2021	• Local protocols for patient management	• **Nutrition target** = Achieve delivery of 60% energy and protein targets<br>• **Reasons for EN interruptions** = fasting for a medical intervention or procedure, EN intolerance, a worsening clinical status, a lack of enteral access or nonavailability of an appropriate formula, or other reasons<br>• **EN intolerance** = as abdominal distension, abdominal discomfort, emesis, and/or diarrhea<br>• **Estimated Energy Requirements** = utilized Schofield equation
Martinez 2023	• ASPEN/Society of Critical Care Medicine (SCCM) 2017<br>• Local protocols for patient management	• **Nutrition goal** = Achieve delivery of 60% energy and protein targets<br>• **Enteral nutrition intoleranc**e = abdominal distension, abdominal discomfort, emesis, and/or diarrhea
Widyastuti 2024	• World Health Organization (no date given)<br>• ESPGHAN/ESPEN/ESPR/CSPEN 2018	• **Estimated energy requirement**s = utilized WHO or Schofield equation<br>• **Daily Caloric Intake Goal=** Achieve 80% of the daily caloric requirement<br>• **Delays in Nutrition Initiation** = due to factors such as shock, GI bleeding, inotropic medication use, and food intolerance<br>• **Protein and fat requirements** = were determined by recommended dietary allowance<br>• **Protein requirement** = 1 gram/kg/day<br>• **Fat requirements** = 1.5 grams/kg/day

		• **Carbohydrates requirement** = remaining calories allocated to carbohydrates
Winderlich 2024	• American Society for Parenteral and Enteral Nutrition (ASPEN) 2009<br>• Institute of Medicine (IOM)<br>• National Health and Medical Research Council	• **Early enteral nutrition** = achieving 25% of the prescribed energy target within the first 48 h of PICU admission<br>• **Estimated energy requirements =** utilized Schofield and Institute of Medicine

Supplemental Table S5. Contents Intervention Type Definitions and Guidelines (n = 11)

First Author Last Name and Year	Guidelines	Definitions
Campos-Mino 2023	ASPEN 2017<br>WHO	• **Caloric Target/Energy Requirements** = utilized the schofield equation<br>• **Protein Target** = achieve a minimum of 1.5 g/kg/d according to ASPEN guidelines<br>• **Nutrition Adequacy =** calculated as 1) the percentage of enteral nutrition delivered for energy and protein referenced to the amount prescribed, and 2) the percentage of enteral nutrition delivered for energy and protein referenced to the amount needed according to international recommendations.<br>• **Malnutrition =** utilizing z scores, definitions of malnutrition were those recommended by the 2015 consensus from ASPEN.
El Koofy 2019	ASPEN 2009<br>WHO	• **Nutritional adequacy** = % of energy and protein goal achieved<br>• Goal energy intake = predicted energy expenditure x stress factor (1.5-2)<br>• **Reasons for feeding interruption** = severe respiratory distress, gastrointestinal bleeding, paralytic ileus, poor adherence of PICU staff to feeding regi-mens were recorded, gastrointestinal intolerance<br>• **Malnutrition** = World Health Organization z scores ≤ -2 standard deviation [SD]<br>• **Severe malnutrition** = z scores ≤ -3 SD
FernandezMontes 2023	American Society for Parenteral and Enteral Nutrition (ASPEN)/Society of Critical Care Medicine (SCCM) 2017	• **Goal energy intake** = 65 kcal/kg/day<br>• **Feed intolerance** = diarrhea, abdominal distension and high residual gastric volume
Haines 2023	*ASPEN 2012<br>*ESPGHAN/ESPEN/ ESPR/CSPEN 2018	-
Hauschild 2019	ASPEN 2009	• **Feed intolerance** = EN interruptions, increased stool frequency, abdominal distension, and increased blood urea and creatinine levels<br>• **Feasibility** = percentage of patients who were eligible, recruited, enrolled, and randomized, and consented and completed the study procedures and follow-up
Marino 2019	ASPEN 2009	• **Malnutrition** = weight for age ≤ −2 Z-scores of the mean of the WHO child growth standards

First Author Last Name and Year	Guidelines	Definitions
	WHO 2015<br>SCCM 2017	• **GI symptoms** = gastric residual volume (GRV) (mL kg−1 day−1), vomiting (frequency) and constipation defined as ≥4 days without stools<br>• **Reasons for withholding feeds** = Mechanical bowel obstruction, suspected necrotizing enterocolitis, significant GI bleed, bowel ischemia, significant abdominal distention, feeding intolerance<br>• **Feeding intolerance** = large GRV, vomiting<br>• **Feed advancement rate for PICU 1** = 0.5–1 mL kg−1 h−1 depending on fluid allowance<br>• **Feed advancement for PICU 2** = Noncardiac diagnosis: 2 mL kg−1 h−1Cardiac diagnosis: 0.6–2 mL kg−1 h−1 depending on fluid allowance
Rooze 2019	Local institutional protocol 2015	• **Feed intolerance** = more than two episodes of emesis or more than four liquid stools per day, insufficient enteral intake to meet energy requirements<br>• **Chronic malnutrition** = H/A below − 2 z-score and acute malnutrition by a W/H below − 2 z-score.<br>• **Energy target** = Larsen et al.: How Energy Intakes Are Associated with Adverse Outcomes in Infants After Open Heart Surgery. 2013
Tan 2021	ESPGHAN/ESPEN/ESPR/CSPEN 2018	• **Metabolic measurements** = β-oxidation of very-long-chain fatty acids, arginine and proline metabolism, pentose phosphate metabolism, ketone body metabolism, citric acid cycle, purine metabolism, caffeine metabolism, pyruvate metabolism, valine leucine/isoleucine/ degradation, and taurine and hypotaurine metabolism<br>•
Tramonti 2018	Clinical practice guideline by Hospital de Pediatría Garrahan 2016	• **Major burn patients** = those who suffer thermal damage affecting more than 30% of total body surface area<br>• **Energy intake** = BMR + 30%<br>• **Acceptable intake** = achieving 70% of the energy and protein target<br>• **Basal Metabolic Rate =** Calculated by the Schofield equation
Winderlich 2024	Local protocol of centers involved 2021<br>No established protocol	• **Increased energy and protein density enteral nutrition support** = fortified expressed breast milk, concentrated infant formula, or ready-to-feed concentrated formula<br>• **Increased energy and protein density oral nutrition support** = receiving fortified expressed breast milk, concentrated infant formula, ready-to-feed concentrated formula, an oral nutrition supplement drink, or a high-energy high-protein diet
Xu 2025	Chinese	• **Feed advancement**= initial volume between 10–20 mL, gradually increased by 5–10 mL If insufficient, parenteral nutrition supplementation, initiated at 0.5–1.0 g/(kg•d) and gradually increased to 3.0–3.5 g/(kg•d)<br>• **Gastrointestinal intolerance** = abdominal distension, diarrhea, and gastrointestinal bleeding
